# A novel one-step lens cleaning device using air and water flow for endoscopic surgery

**DOI:** 10.1371/journal.pone.0200749

**Published:** 2018-07-18

**Authors:** Hironori Tatsuki, Takehiko Yokobori, Chika Katayama, Ryuji Kato, Ryo Takahashi, Katsuya Osone, Takahiro Takada, Reina Yajima, Yoko Motegi, Hiroomi Ogawa, Takaaki Fujii, Ken Shirabe, Hiroyuki Kuwano, Takayuki Asao

**Affiliations:** 1 Department of General Surgical Science, Gunma University, Graduate School of Medicine, Maebashi, Gunma, Japan; 2 Big Data Center for Integrative Analysis, Gunma University Initiative for Advance Research, Maebashi, Gunma, Japan; Osaka Medical Center for Cancer and Cardiovascular Diseases, JAPAN

## Abstract

In a surgical operation requiring endoscopy, it is essential to obtain a clear endoscopic view. However, it is often disturbed by the contamination on the lens during the surgery. No device can clean the lens surface simply and completely. Many surgeons are hampered by the impaired view and the distraction by the repeated cleaning of the lens. Therefore, we developed a novel endoscope cleaning device to address this problem. The device was made of 3D-printed rubber-like plastic. It contains a syringe filled with saline and an aspiration system. It would be used intraoperatively to wash the lens surface in a few seconds with rapid flow of water and air. The cleaning ability of the device was evaluated using mayonnaise with adenosine triphosphate (ATP) as a model contaminant. The gauze-wiping maneuver was selected as control. After each maneuver, the clarity of the endoscopic view was evaluated, and residual contaminants were assessed quantitatively with ATP assay. The cleaning device obtained a crisp and clear view and eliminated the contaminant on the lens every time after a single cleaning maneuver. The gauze-wiping maneuver required for the lens to be wiped at least three times to obtain a clear view, and even then, some contaminants remained. Repeated contamination and cleaning using gauze led to accumulation of contaminants on the lens, which resulted in difficulty in cleaning the lens as the operation proceeded. The cleaning device did not show such accumulation. Our novel cleaning device with air and water flow has been shown to wash out the lens contaminants completely and immediately in a simple manner. It is expected to improve the safety and cost-effectiveness of endoscopic surgery.

## Introduction

Endoscopic surgery has become widely accepted as a precision, minimally invasive surgery. The operation depends strongly on the endoscopic view and requires more skills and concentration of the surgeon than that in open surgery. Therefore, many improvements to the endoscope and related equipment have been made.

However, the endoscopic view is often easily impaired by water condensation or contamination by mists of body tissue or fluid. These mists contain proteins and fats, which tend to adhere to the lens surface on the endoscope tip. Although repeated cleaning of the lens is required during procedures, which is time consuming and distracting, there have been few improvements in lens cleaning methods. Yong et al. reported that 37% of operative time was spent under impaired endoscopic views and 7% was used for lens cleaning during endoscopic operations by surgeons [1]. Moreover, most surgeons are reluctant to clean the lens and sometimes continue the operation with contaminated lens, which is unfavorable from a safety viewpoint.

The most common way to clean the endoscope lens is to wipe the scope tip with gauze. This requires time to wipe thoroughly, and the achieved view is not always sufficiently clear. Additionally, operative time and stress for the surgeon may be increased. Some methodologies to avoid fogging, contamination, or cleaning the lens have previously been reported [2]. Some commercial devices can provide or maintain a clear endoscopic view [3,4]. In actual practice, however, most of these devices are not commonly used [1]. Considering this situation, it would be useful to have an alternative method of cleaning the endoscopic lens completely in one short action of a novel device. Moreover, such a device should be inexpensive and simple enough to be prepared in an operation room.

The purpose of this study was to develop a novel endoscopic lens cleaning device that could be conveniently used in the operating room. We compared the endoscopic views and cleaning efficiencies between the device and the conventional gauze-wiping method. Our novel cleaning device was designed to wash away lens contaminants immediately and completely without any special equipment or specific techniques, which may contribute to the safety and cost-effectiveness of endoscopic surgery.

## Materials and methods

### Three-dimensional modeling and construction of the novel lens cleaning device

**Fig 1:**
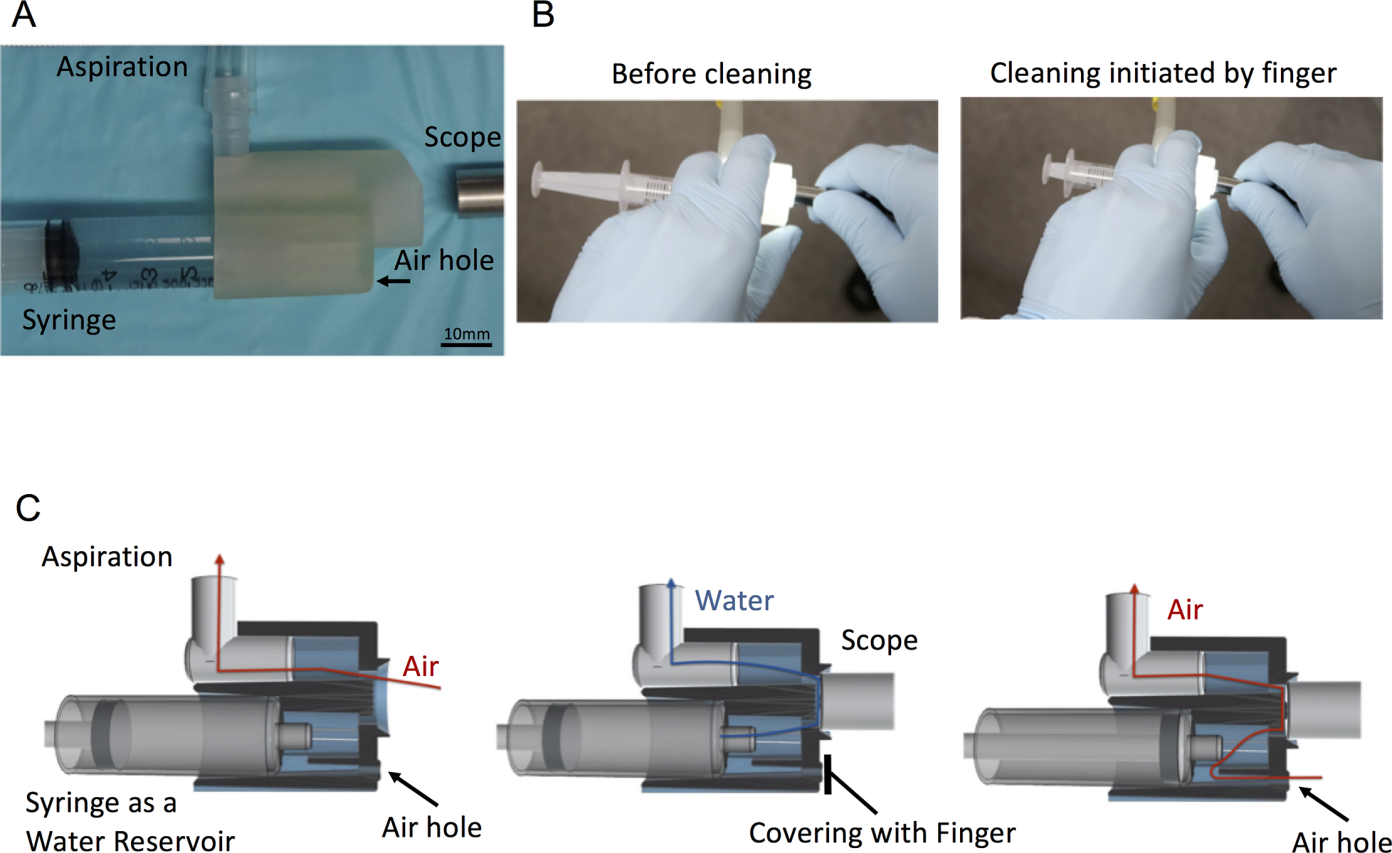
Mechanism and structure of our novel lens cleaning device. A. Appearance. B. Manipulation techniques for lens cleaning started by covering the air hole with a finger. C. Schema of the device. The left panel shows that the air runs through the device without covering the hole with a finger, the middle panel shows the water cleaning initiated by air-hole covering with finger and scope, and the right panel shows that water drops on the lens surface after cleaning are removed by air aspiration through the air hole.

The three-dimensional structures of the cleaning device were designed using the Autodesk 123D design tool system (Autodesk, North Ryde, Australia). The 3D structural data in standard triangulated language format were extracted from the designing software, and a commercial 3D printing service, DMM.make (http://make.dmm.com/print/), was employed for the actual manufacturing of the model. A rubber-like plastic was selected as the construction material. A 3D structural schema of our cleaning device is shown in Fig 1C.

### Quantitative analysis of residual contaminants by adenosine triphosphate luminescence assay

We selected mayonnaise as a model contaminant. Adenosine triphosphate (ATP) was suspended in mayonnaise for quantitative analysis of the residual contaminant on the lens. A 50-μl aliquot of mayonnaise was placed on the endoscopic lens at each evaluation of cleaning methods. Lumitester PD-30 (Kikkomanbiochemifa Corp., Tokyo) was used to evaluate the residual ATP according to the manufacturer’s protocol.

### Assessment of endoscopic view

To compare the clarity of the endoscopic view, we captured a digital still image with the scope under each condition. A bunch of artificial flowers was set in a dark box as a subject. A small LED light source was also set in the subject to assess slight impairment of the clinical endoscopic view with the diffused reflection on the lens surface, which may cause flares or a hazy view around the image when strong light is applied in the model view.

### Gauze-wiping and novel device cleaning methods for endoscopic lens

We chose a specific gauze-wiping maneuver, which was considered the most common method used in clinics, as a control cleaning method. In the gauze-wiping maneuver, we rubbed the scope tip thoroughly with a single side of a non-woven cotton gauze square (12.5 cm × 12.5 cm). All gauze-wiping maneuvers were performed by the same surgeon who is experienced in endoscopic surgery. In the device maneuver, 5 ml of saline was placed in the syringe, and the scope was cleaned with aspiration in a second. The cleaning maneuver was repeated three times to assess cleaning effectiveness.

### Statistical analysis

Differences in relative light units between our device and gauze wiping were estimated by performing Student’s t-test. Differences were accepted as statistically significant at the level of P < 0.05. Statistical analysis was performed by using JMP software (SAS Institute, Cary, NC).

## Results

### Manipulation of our novel lens cleaning device

Fig 1A shows our lens cleaning device. This device was designed to clean the lens surface by air and water flow. A 5-ml syringe is attached as a water reservoir. The water content is aspirated by covering the air hole with a finger, which causes a vacuum in the water channel and washes the contaminants off. When the finger is released, the suctioned air flow removes the water drops on the lens thoroughly (Fig 1B and 1C). Saline, instead of pure water, was used in the experiments because it may be easily available in an operation room.

### Lens surface cleaning was more efficient using the tested device than using gauze wiping

To show how efficient our novel device could clean the lens, we used mayonnaise with ATP as a model contaminant. In addition to estimating the clarity of the scope view, we measured the residual amount of ATP on the lens after each cleaning maneuver to quantitatively estimate the cleaning efficiency. We commonly clean the lens by wiping off the contaminant with gauze, so the cleaning efficiency of the device was compared to the gauze-wiping maneuver.

**Fig 2:**
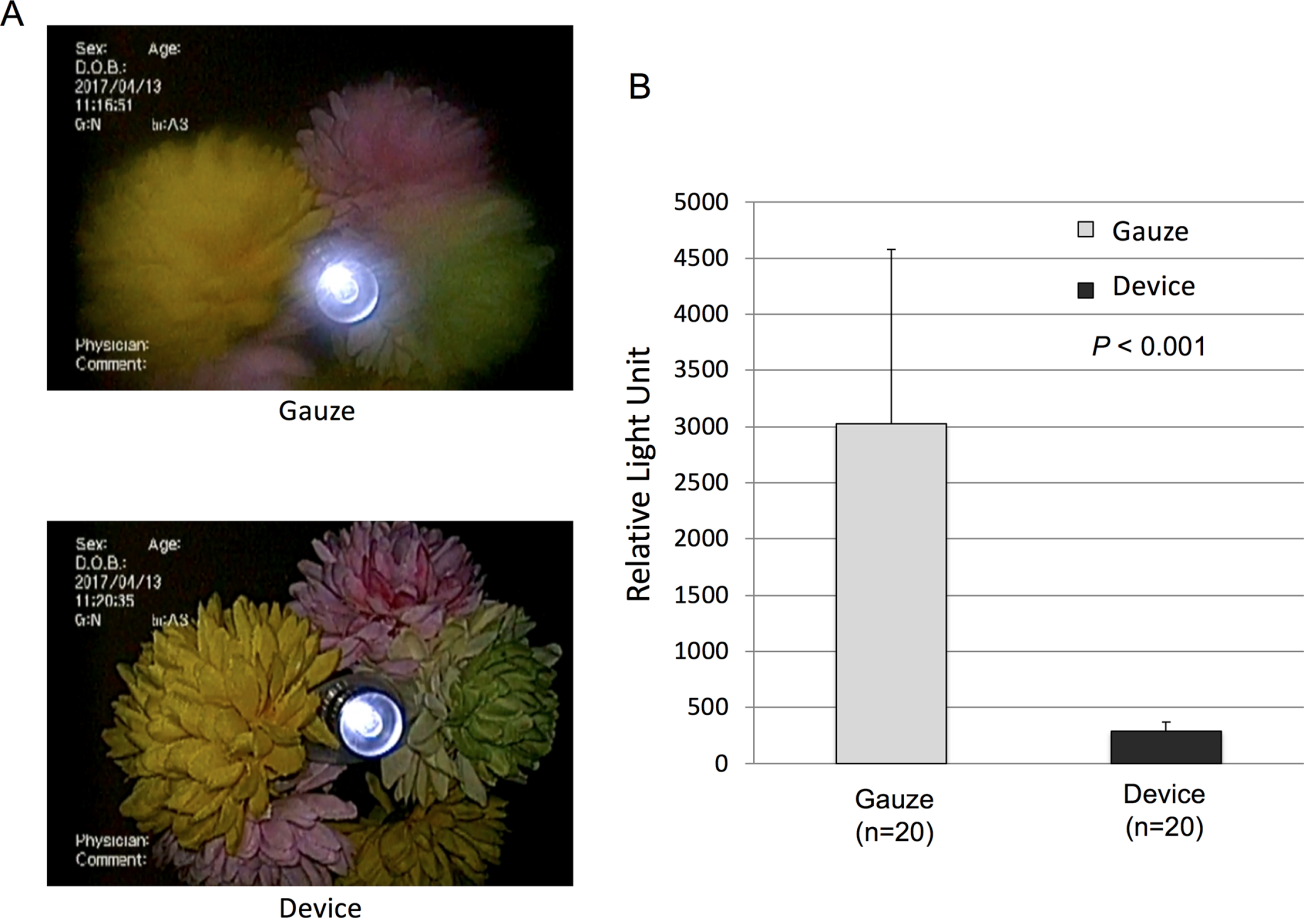
Our device had better cleaning ability than that of gauze wiping. A. Representative endoscopic views: the upper panel shows the image after cleaning the contaminated lens with gauze, and the lower panel shows the contaminated lens after cleaning with the device. B. Luminescence evaluation of residual contaminants on the lens after cleaning by gauze and by the device.

The cleaning efficiency in each single cleaning maneuver was evaluated. Although the scope view was still blurry in most cases when the lens was wiped only once with gauze, it was crisp and clear every time after cleaning with the test device (Fig 2A). The amount of residual contaminant also showed the same tendency (Fig 2B), and the amount of residual contaminant was consistently 10 times lower after using the test device than after gauze wiping.

### Impaired endoscopic view was cleared in one step by using our device

**Fig 3:**
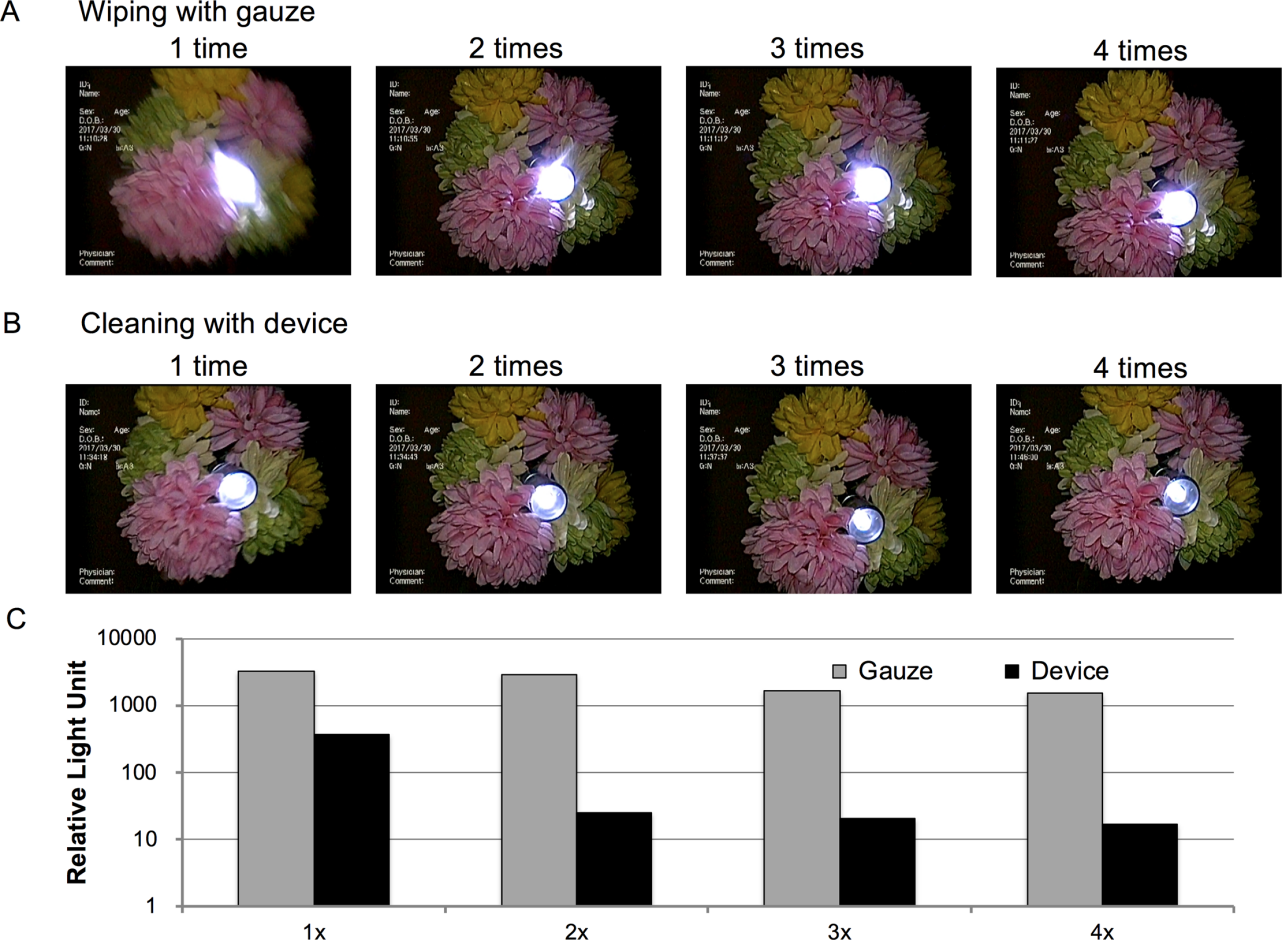
Washing effectiveness of the gauze and device. A. Representative images of the endoscopic view after repeated cleaning of the contaminated lens after gauze wiping. B. Representative images of the endoscopic view after repeated cleaning of the contaminated lens according to the number of device cleaning. C. Luminescence evaluation of residual contaminants on the lens after cleaning according to the number of cleaning by gauze and the device.

To determine how many gauze-wiping attempts were needed to achieve a clear view, residual contaminant was measured after successive gauze-wiping attempts (Fig 3). The results showed that after three attempts, an acceptable clear view could be achieved, but the clarity did not improve further after the fourth attempt. These observations were confirmed quantitatively (Fig 3C).

The clarity of the scope view was assessed by looking for flares and haziness around the image of the light source, which are caused by diffuse reflections on the dirty lens surface after gauze wiping (Fig 3A). After using the test device, the view was sufficiently clear after the first maneuver (Fig 3B).

### Repeated cleaning with gauze wipes led to accumulation of contaminants on the lens surface

**Fig 4:**
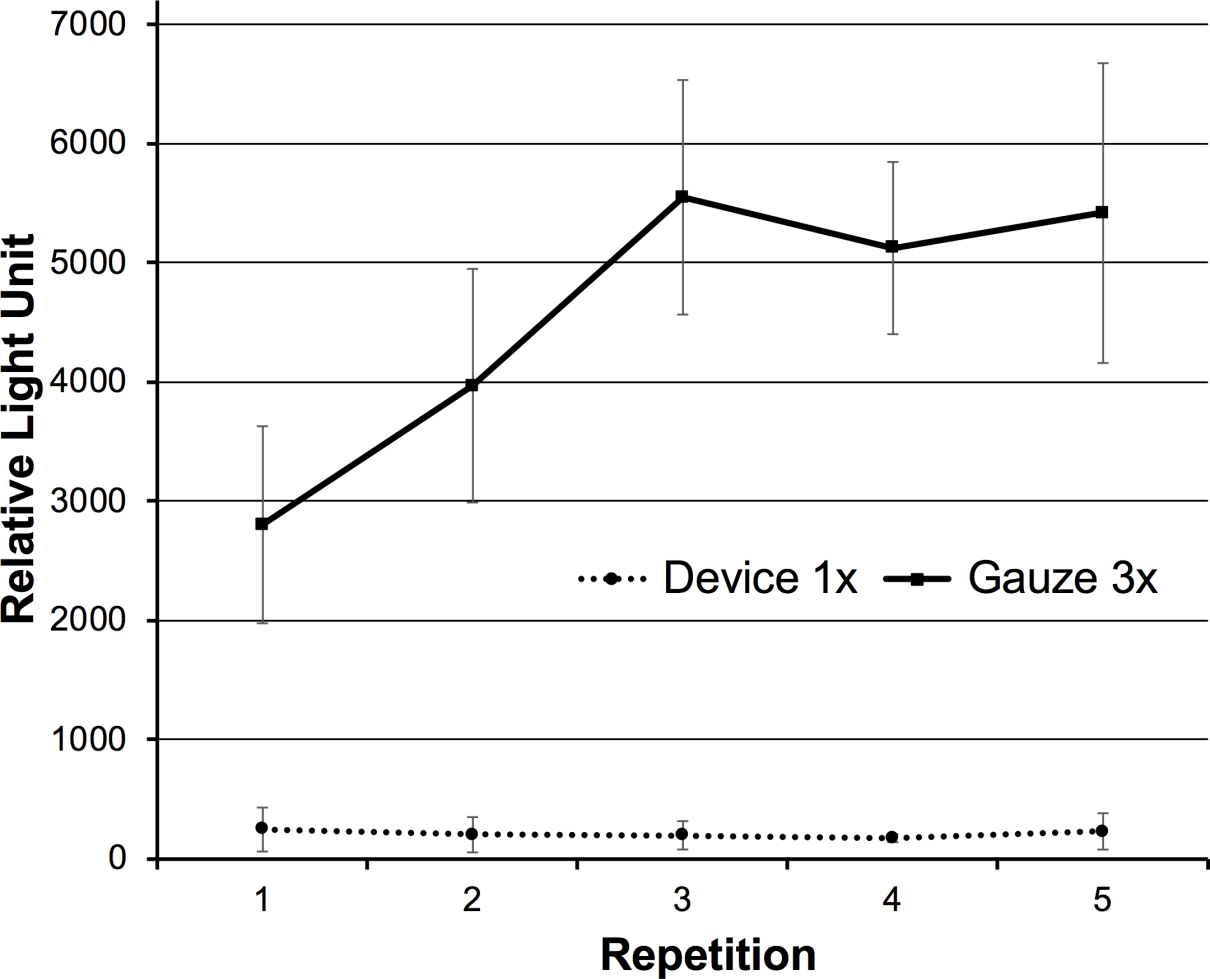
Accumulation of contaminants on the lens surface after repeated cleaning. Three wipes with gauze or one cleaning pass by using our device were performed repeatedly.

Frequently, the endoscopic lens becomes more easily contaminated as the operation proceeds. We assumed that this was because some of the contaminants accumulated on the lens surface when the lens was cleaned only by repeated gauze wiping. By repeating the contamination and cleaning steps, we demonstrated that accumulation did occur (Fig 4). We determined that accumulation occurred after each gauze-wiping pass. No such accumulation occurred when using the test device.

## Discussion

In this study, we demonstrated that our novel cleaning device could effectively clean the endoscopic lens. Our data showed that the device washed the test contaminants off the lens quickly and easily. Repeated gauze wiping, which is the most common procedure for lens cleaning in clinics, caused contaminant accumulation on the endoscopic lens. Our novel device cleaned the lens contaminants consistently and completely in one action without accumulation.

Impaired endoscopic views are well-known stressors for surgeons in endoscopic surgery. Impaired views and repeated cleanings increase the operative time and are at risk for surgical errors. Therefore, it is essential to maintain a clear view through the scope in endoscopic surgery.

One of the causes of blurred vision is fogging. Fogging mostly occurs at the moment the endoscope is introduced into the cavity. Water from the warm and humid air in the body cavity condensates on the lens surface when the scope tip is colder. To avoid condensation, warming the scope tip in a hot water bath and immersing in an anti-fogging solution are popular methods, for which improvements have been suggested [5–7]. Another cause is that the contaminants come from body tissues or fluids. Contamination occurs by accidental contact with the visceral wall or from the mist scattered in the air from coagulation devices.

Once the contaminants are attached to the solid glass lens surface, they are difficult to remove completely. Because many contaminants contain proteins and fats, wiping with dry gauze spreads the contaminants, which then dry to form a thin layer of contaminants on the lens surface. These mechanisms make removal using the gauze-wiping method difficult. Several wiping passes are needed to obtain a clear view, but some of the contaminants remain on the surface, which leads to accumulation after repeated wiping. As the operation proceeds, the lens can become contaminated more easily and more difficult to be cleaned thoroughly.

A better way to clean the lens surface thoroughly is to let the contaminants rise from the surface and blow them off with rapid water flow. Our device has a unique functional design that generates a rapid and turbulent flow of water across the lens surface. To start the turbulent flow of the water and air just before the lens, the device was designed to be attached in an air-tight manner to the scope, and it has a wavy surface to make contact with the tip surface of the scope. By covering the air hole, water from the syringe runs across the surface of the scope and blows off the contaminants. The operator can control the flow by the timing and frequency of covering the hole with a fingertip. By keeping the device hole uncovered, residual water drops can be removed by air flow. This finally dries the tip of the scope.

In this study, we have demonstrated that this mechanism works well and removes contaminants consistently and completely. Moreover, the required manipulation is very simple that anyone can obtain the same cleaning effect. Clinically, one limitation of our device is that it is designed for cleaning, not as an anti-fogging device. The rapid flow of water and air may draw some heat from the lens surface, which sometimes causes fogging. This problem could be overcome by using warm (42 degrees centigrade) water and keeping the last drying step abrupt.

Our device is also easy to set up and small enough to place immediately outside the operative field. The device only requires an aspiration tube and a syringe filled with saline, which are commonly available in most operation rooms. The structure is simple and can be manufactured inexpensively. Therefore, this device may be easily introduced into many operation rooms at a very low cost and effort.

It would be useful if a cleaning device could be used intra-abdominally or intra-thoracically. Such device may save time and reduce the risks associated with removing and re-introducing the endoscope [8]. Some commercial devices for this purpose are available. One device is a cleaning fabric that is released and anchored in the cavity [3,4]. The area of the cloth fabric is small but has the same limitations as those of the wiping method. Another is a sheath-shaped device, which is attached to the tip of the endoscope. When using this device, the lens can be effectively washed with a flow of water, but the drainage is wasted inside the body cavity. The structure is complex; therefore, it is non-reusable and relatively expensive. Similarly, our device is disposable, but simple enough to be integrally molded with plastic. Since it is designed to be used outside the body cavity, it does not affect the cavity internally. By mass production, our device could be produced at a low cost at approximately 10 USD (including the sterilizing cost).

Many medical professionals who work in operating rooms have long desired for a low-cost endoscopic lens cleaner with high washing efficiency that is disposable and can be quickly and easily manipulated. We believe that this device provides these benefits.

## Conclusions

We developed a novel endoscopic lens cleaning device and demonstrated that it could clean the lens more effectively than using the conventional gauze-wiping method. The device can be easily introduced in the operation room and helps to maintain a clear view in endoscopy. By using this device, surgeons can concentrate better on the surgical procedures and will have a longer duration of clear view. These benefits may help shorten operative times and improve the safety of endoscopic surgery. To determine the usefulness of our device in a greater number of situations, clinical trials are expected to be conducted in the future.
